# Genome Sequences of 12 Cluster AN *Arthrobacter* Phages

**DOI:** 10.1128/genomeA.01092-17

**Published:** 2017-11-09

**Authors:** Julia Y. Lee-Soety, Shantanu Bhatt, Tamarah L. Adair, J. Alfred Bonilla, Karen K. Klyczek, Melinda Harrison, Saint Joseph’s University SEA-PHAGES, Baylor University SEA-PHAGES, University of Wisconsin-River Falls SEA-PHAGES, Cabrini University SEA-PHAGES, Rebecca A. Garlena, Charles A. Bowman, Daniel A. Russell, Welkin H. Pope, Deborah Jacobs-Sera, Steven G. Cresawn, Graham F. Hatfull

**Affiliations:** a Department of Biology, Saint Joseph’s University, Philadelphia, Pennsylvania, USA; b Department of Biology, Baylor University, Waco, Texas, USA; c Biology Department, University of Wisconsin-River Falls, River Falls, Wisconsin, USA; d Department of Science, Cabrini University, Radnor, Pennsylvania, USA; e Department of Biological Sciences, University of Pittsburgh, Pittsburgh, Pennsylvania, USA; f The Scripps Research Institute, La Jolla, California, USA; g Department of Biology, James Madison University, Harrisonburg, Virginia, USA

## Abstract

Twelve siphoviral phages isolated using *Arthrobacter* sp. strain ATCC 21022 were sequenced. The phages all have relatively small genomes, ranging from 15,319 to 15,556 bp. All 12 phages are closely related to previously described cluster AN *Arthrobacter* phages.

## GENOME ANNOUNCEMENT

*Arthrobacter* spp. are soil bacteria useful for bioremediation and their ability to metabolize hydrocarbons (1, 2). The more than 40 bacteriophage genomes of *Arthrobacter* hosts are diverse, forming 10 clusters (AK to AU) and 2 singletons (3). Ten cluster AN phages previously described are of interest in that they each have a relatively small genome (15.3 to 15.6 kbp) for phages with a siphoviral morphology (3). They are closely related to each other at the nucleotide level, even though they were isolated in geographically distinct locations (3).

We have isolated and characterized 12 bacteriophages that infect *Arthrobacter* sp. strain ATCC 21022 (4). All the phages were isolated from soil samples by students in the Science Education Alliance-Phage Hunters Advancing Genomics and Evolutionary Science (SEA-PHAGES) program (5) at nine different locations (Table 1) using an enrichment procedure, except for phage Lore, which was isolated by direct plating. They have a siphoviral morphology with a relatively small isometric head (39 ± 7 nm diameter) and a noncontractile tail (98 ± 15 nm).

**TABLE 1:** Cluster AN *Arthrobacter* phages

Phage name	GenBank accession no.	Genome length (bp)	G+C content (%)	No. of genes	Location of isolation
Chestnut	KY434670	15,556	60.1	26	Radnor, PA
Courtney3	KX443695	15,556	60.1	26	Katy, TX
Elkhorn	MF140409	15,556	59.6	26	Waco, TX
KylieMac	MF140415	15,540	59.8	27	Waco, TX
Link	MF140417	15,521	60.2	26	Waco, TX
Lore	MF140419	15,556	59.6	26	Waco, TX
Mariposa	MF140420	15,556	60.1	26	Downingtown, PA
Massimo	KX576642	15,556	60.1	26	Camp Hill, PA
Prospero	KX610765	15,556	60.1	26	Vineland, NJ
Seume	MF140426	15,319	60.3	26	Somerset, WI
Taj14	MF140431	15,546	59.9	26	Missoula, MT
TinoCrisci	MF140433	15,556	60.1	26	Devon, PA

Phage genomes were sequenced using the Illumina MiSeq platform at either the North Carolina State University Genomic Sciences Laboratory or the Pittsburgh Bacteriophage Institute using 150-bp unpaired reads. Sequences were assembled using Newbler, generating major contigs with coverage from 2,032- to 12,826-fold. The genomes are similarly sized (15.3 to 15.6 kbp) with similar G+C content (~60%), and all have a defined end with 11-base 3′ single-stranded DNA extensions (right end, 5′-CCCGCGCCACC) (Table 1). All of the phages are closely related to other cluster AN phages (6), with >85% pairwise average nucleotide sequence identities, spanning >95% of their genome lengths. Genomes were annotated using DNA Master (cobamide2.bio.pitt.edu) with coding sequences predicted by GeneMark (7) and Glimmer (8), and 26 to 27 protein-coding genes were identified (Table 1). No tRNA or transfer-messenger RNA (tmRNA) genes were detected by Aragorn (9) or tRNAscan-SE (10).

Except for a single leftward-transcribed gene in each genome (e.g., Courtney3 *21*), all the genes are transcribed rightward. These include virion structure and assembly genes, including the terminase large subunit, portal, tape measure protein, minor tail protein, and fused capsid-protease genes. These are followed by a lysis cassette in which peptidase and amidase functions are encoded by two separate genes, and four genes coding for predicted DNA binding proteins with predicted helix-turn-helix DNA binding motifs, and an HNH endonuclease. We did not identify genes coding for DNA replication or DNA metabolism functions, and it is unclear how replication is initiated or regulated. The primary difference in gene content among the 12 phage genomes is a small gene (e.g., Courtney3 *2*) located near the genome left end that is absent from phages KylieMac, Seume, and Taj14. None of the phages encode integrases or partitioning systems, and there is no evidence that any form stable lysogens. Although we predict that a programed translational frameshift plays a role in expression of the tail assembly chaperones—a well conserved feature of the *Siphoviridae* (11)—the position of the putative frameshift is not readily apparent from bioinformatic analyses.

### Accession number(s).

GenBank accession numbers are provided in Table 1.
